# Effects of solid dispersion of curcumin in metabolic and immunological alterations during experimental sepsis

**DOI:** 10.1186/cc12999

**Published:** 2013-11-05

**Authors:** Letycia Silvano da Silva, Tatiana Tocchini Felippotti, Gabriela Ravanelli de Oliveira Pelegrin, Luis Henrique Angenendt da Costa, Sérgio Olavo Petenusci, Luiz Alexandre Pedro de Freitas, Maria José Alves da Rocha

**Affiliations:** 1 Department of Morphology, Physiology and Basic Pathology, Dental School of Ribeirão Preto, University of São Paulo, Ribeirão Preto, Brazil; 2 Department of Pharmaceutical Sciences, Faculty of Pharmaceutical Sciences of Ribeirão Preto, University of São Paulo, Ribeirão Preto, Brazil

## Background

Studies suggest that curcumin, found in the tropical plant *Curcuma longa*, has anti-inflammatory and antioxidant properties and can act in sepsis, decreasing the release of proinflammatory mediators and free radicals. In the search to increase curcumin's bioavailability a fitotecnologic process was developed that generated a solid dispersion of curcumin named DS17. This dispersion is water soluble and seems to increase the curcumin absorption by the gastrointestinal tract. The aim of our study was to assess the biological activity of the solid dispersion of curcumin (DS17) in immunological and metabolic alterations observed in a model of sepsis in rats induced by CLP.

## Materials and methods

Male Wistar rats (250 to 300 g) were divided into two groups: polymicrobial sepsis model by cecal ligation and puncture (CLP) and sham operation (OF). The animals were pretreated with DS17 (100 mg/kg) orally for 7 days prior to CLP and treated 2 hours after surgery. The animals were used to analyze curcumin absorption through HPLC, plasma glucose, cytokines, nitric oxide (NO) and HSP70. Another group had the survival rate assessed for 48 hours.

## Results

Our results showed that curcumin is present in the plasma at 4 and 6 hours but absent 24 hours following the DS17 administration. The dispersion decreased IL-6 in plasma and peritoneal fluid at 6 and 24 hours, and IL-1β 6 hours after sepsis stimulus. Moreover, we observed an increase in the hematocrit and a decrease in plasma glucose in the same animals. Paradoxically, plasma IL-10 and serum HSP70 decreased in 24 hours while plasma NO increased in the same period. These changes were not sufficient to increase significantly the survival although we observed a biological improvement of 20% 24 hours following CLP.

## Conclusions

Our results suggest that despite a significant decrease in proinflammatory cytokines (IL-1β and IL-6), treatment with curcumin solid dispersion produced no beneficial biological effect in septic animals. Further studies are necessary to better clarify the suggested antioxidant and anti-inflammatory effect of curcumin.

